# Factors associated with differences in perceived health among German long-term unemployed

**DOI:** 10.1186/1471-2458-12-485

**Published:** 2012-06-27

**Authors:** Heribert Limm, Mechthild Heinmüller, Katrin Liel, Karin Seeger, Harald Gündel, Ahmet Kimil, Peter Angerer

**Affiliations:** 1 Department of Psychosomatic Medicine and Psychotherapy, University Hospital of Ulm, Am Hochsträß 8, D-89081, Ulm, Germany; 2 Department of Occupational, Social and Environmental Medicine, Ludwig-Maximilians-University, Ziemssenstr. 1, 80336, Munich, Germany; 3 Ethno-Medical Center e.V. (EMZ), 30169, Hanover, Germany; 4 Institute for Occupational Medicine and Social Medicine, Heinrich Heine University Düsseldorf, Universitätsstr. 1, 40225, Düsseldorf, Germany

**Keywords:** Long-term unemployed, SF-12, HADS-Anxiety, HADS-Depression, Self-perceived health

## Abstract

**Background:**

Unemployment is associated with reduced physical and psychological well-being. Perceived health is an important factor influencing health outcomes as well as successful returns to work. This study aims to determine the extent to which perceived health correlates with mental health, various health risk characteristics and socio-demographic characteristics in a setting-selected sample of long-term unemployed persons.

**Methods:**

Using SF-12, 365 long-term unemployed persons were assessed for self-perceived health and various socio-demographic and health characteristics. Perceived health data of the sample was compared to the German SF-12 reference population. Bivariate analyses and multiple linear regression models were applied to identify those variables significantly associated with perceived health.

**Results:**

The study population reported poorer perceived health compared with the general population. Analyses showed that perceived mental health was significantly worse in women, among persons with heightened depression and anxiety scores, and in participants reporting reduced levels of physical activity. Perceived physical health was significantly lower among older persons, participants with a higher BMI, and participants with heightened depression and anxiety scores. Both mental and physical health were worse among the unemployed assigned to an employment center as compared to those engaged in the secondary labor market. In total, 36% of the variance in the SF-12 mental score and 20% of the variance in the SF-12 physical score were explained by the factors included in the final multiple linear regression models.

**Conclusions:**

Perceived health among a select group of long-term unemployed is reduced to a clinically relevant extent compared to the general population. The preliminary findings underline an association between mental health and perceived health. Negative self-perceptions of health were also associated with the labor market setting and some of the socio-demographic and health behavior variables. Further research is needed to determine risk factors leading to reduced perceived health in the unemployed. The strong association between mental health and perceived health suggests interventions targeting mental health are urgently needed to positively influence perceived health, a key determinant of individuals’ chances to successfully return to work.

## Background

Unemployment is a known health hazard. Since the first studies on the effects of unemployment [1], a growing body of evidence has developed showing that job loss is associated with negative objective and subjective health [2-7] and increased levels of morbidity and mortality [8]. Factors associated with health differences among the unemployed, especially among the long-term unemployed, require further examination however, since this group remains a primary target of re-employment efforts.

Adverse health outcomes, such as myocardial infarction, hypertension and increased alcohol and nicotine consumption, are common among the unemployed [9]. Unemployed persons are more likely to be on medication, need more medical consultations, and have longer and more frequent hospital stays [9-11]. Interestingly, one of the most extensively studied health effects of unemployment is the decreased level of psychological well-being among the unemployed [12,13].

A recent meta-analytic investigation reported an estimated 34% of unemployed persons carry some form of psychological problem, as compared to 16% among the employed population [13]. Brähler et al. [14] identified higher levels of anxiety, depression and physical complaints in three representative samples of unemployed persons in Germany as compared with the employed population. Dooley et al. [15] analyzed the American National Longitudinal Survey (NLS) and found that unemployment led to significant increases in incidents of depression. In a major Australian study, the 1993 National Survey of Mental Health and Well-being, 22% of unemployed persons reported symptoms of depression, as compared to 5% reporting such symptoms in the general population [16]. On the positive side, exit to paid labor has been shown to improve mental well-being [17].

The relationship between unemployment and poorer health is bi-directional and the selection causation mechanisms are mediated by other variables, such as socioeconomic factors, which can include unemployment rate, health behavior and psychosocial variables. According to Artazcoz et al. [12], the effects of unemployment on mental health are not equally distributed across gender and family roles, or across social class categories. In their meta- analysis, Moser and Paul [18] found that mental health in particular is negatively affected by long-term unemployment—defined as having been unemployed for more than one year—being male, having attained a lower level of education, and by age, with the young being affected disproportionately. A separate meta-analysis of 104 empirical studies showed that education, gender, unemployment duration, and sample type—school drop-out vs. mature unemployed—moderated the relationship between mental health and unemployment. The longer the person had been searching for a job, the more likely the individual was to experience depression. The authors also emphasize the importance of the individual’s cognitive appraisal of the job loss as an important component of well-being and health for the unemployed [4].

Perceived health has been shown to be a reliable predictor of total mortality, psychological and medical symptoms, as well as return to work [13,19,20]. In several unemployment studies, perceived health was considered to be an important target of rehabilitation and intervention efforts [20-22], in addition to more objective outcomes, such as symptom relief and physical health. Self-rated health is often mentioned as a potential barrier to (re)employment, and is thus a primary goal in health promotion programs for the long-term unemployed [22]. Patients’ own perceptions of health and ability to work have been shown to be relevant predictors of an eventual and successful return to work in rehabilitation trials [23] and following myocardial infarction [24].

Evidence regarding the relationship between unemployment and self-perceived health is contradictory. Some studies reported that the proportion of persons who self-rate their health as poor are significantly higher in the unemployed population than in the employed population [19]. Other studies show that self-rated health among the unemployed is similar to that of employed persons [21]. Re-employment has been shown to positively influence mental and physical perceived health within a short window of time [20].

These results show that unemployment is clearly associable with harmful health effects. There seems to be great variability, however, in the subjective experience of unemployment and its consequences for the health of individual’s [19,25-27]. Perceived health as a relevant factor for a return to work has received growing recognition as a key point for intervention studies in samples of unemployed persons. A better understanding of the factors associated with perceived health among the long-term unemployed is of paramount importance for the design of suitably effective intervention programs aimed at reducing health inequalities and improving the quality of life for the long-term unemployed. In this study, our aim was thus to more closely examine the differences in perceived health among a setting-selected sample of long-term unemployed individuals.

## Methods

### Overview of design, procedures and recruitment of participants

A sample of long-term unemployed persons in Munich and in Hanover was recruited for a setting-based health promotion programme, sponsored by the Federal Ministry of Education and Research. The data presented in this paper is a cross-sectional analysis of the total sample at baseline. In Hanover, the unemployed were recruited directly at the employment center (job center). The Hanover job centers counseled primarily unemployed persons over 50 years of age. Participants in Munich were recruited from non-profit organizations participating in the secondary labor market. The setting in Munich offered more structure and support for the unemployed than did the Hanover job centers. Participants were included in the study according to the following criteria: between 18 and 65 years of age; entitlement to unemployment benefits according to German SGB-II regulations; and, possessing at least basic proficiency in spoken German. Application for early retirement or severe language barriers led to exclusion. Recruitment lasted from September 2009 through July 2010. In both settings, invitation to participate in the study was disseminated by professionals already engaged in those settings; in many instances, these professionals were already responsible for re-integration activities. The invitation and recruiting process was not standardised, due to setting-specific needs and concerns. Selection effects among eligible participants were inevitable. Information sessions were provided by the study team for all participants at their setting. The evaluation study currently under discussion was carried out by the research group. Participation was entirely voluntary. As motivation to participate, a small pecuniary compensation (10€ for each time point) was offered. In total, 418 unemployed persons agreed to participate in the study. Of that population, 53 interested persons failed to meet the base criteria for inclusion. Thus, 365 participants (87.3% of interested parties) were included in the study. To form a baseline for subsequent comparative study, all participants were required to complete three questionnaires in a group session. The questionnaires were administered by a trained member of the study team at the setting. In the case of language difficulties, the questionnaires were read to the group and explained, if necessary. For specific ethnic groups a translated version of the questionnaire, e.g. in Russian or Turkish language, was made available. In Munich, this data collection process was made a part of regular re-integration activities; in Hanover, participants were asked to come to the job center for that occasion only. In addition, all participants were invited to a 30 minute medical examination. As part of the examination, participants received direct feed-back of the individual results and preventive recommendations by the attending physician. Written, informed consent was obtained, and information was provided in accordance with the Declaration of Helsinki [28]. The Ethical Committee of the University of Munich approved this study.

### Medical diagnostics and assessment of health risk factors

The medical examination included questions on health behavior—i.e. smoking habits, alcohol consumption (AUDIT C), nutritional habits, and physical activity—and preventive care utilization. Height and weight were measured in light clothing without shoes, and the corresponding body mass index (BMI) was calculated (kg/m^2^). Two blood pressure measurements at the upper arm were taken using a fully automatic oscillometric device after the participant had rested for five minutes in a seated position. Of these two measurements, that with the lower systolic value was used in subsequent analysis. Hypertension was defined as systolic blood pressure >140 mmHg, or diastolic blood pressure >90 mmHg. A BMI ≥30 was classified as obese.

All participants received a face-to-face medical consultation, encouraging them toward a healthy lifestyle and—in the case of physical or psychological symptoms or signs of distress—to the use of appropriate health and mental health care services.

### Socio-demographic and unemployment specific data

Socio-demographic data and questions concerning migratory status and duration of unemployment were recorded. Migration experience was defined as personal migration experience—that is, said participant was not born in Germany. Educational level was assessed based on the number of years of school attendance. Participants were also asked to provide information as to whether or not they were part of a steady relationship. The “setting” variable describes whether the participant was recruited directly at an employment center (Hanover) or from the secondary labor market (Munich).

### Assessment of self-perceived health

Self-perceived health was measured with the SF-12 functional health status instrument, an abbreviated form of the Short Form 36 Health Survey (SF-36) [29]. Weighted summation provided summary scores for perceived mental health (MCS = mental health component score) and perceived physical health (PCS = physical health component score). Of the twelve total items, eight refer directly to functional limitations due to health. The SF-12 measurement has been standardized according to US norm data [29], with a mean score of 50 (with a standard deviation [SD] 10). For the German data-set under investigation, the following gender-specific reference scores have been reported: male population PCS = 50.22; MCS = 53.25; female population PCS = 47.93; MCS = 51.30 [30]. The SF-12, which has been used primarily in clinical environments for disease-specific health status monitoring [31], can also be used to measure the relationship between physical and mental health functioning and the social determinants of health. The physical component score (PCS) and mental component score (MCS) for our sample were computed using the algorithm provided with the SF-36-manual [29]. For observations with missing data on a subset of the SF-12 items, a modified version of this algorithm was used to implement the imputation method suggested by Perneger [32]: if at most three of the respective score’s key items were missing, the contribution of each missing item to the overall score (prior to norms-based standardization) was imputed by the mean value at baseline of this item’s contribution (i.e., by weighted summation of the corresponding indicator variables) averaged across all observations containing complete SF-12 data.

### Assessment of anxiety and depression

Anxiety and depression were assessed using the Hospital Anxiety and Depression Scale (HADS) [33]. This scale is a widely used instrument designed to measure psychological morbidity. The HADS questionnaire contains 14 items and results in scores for two sub-scales: anxiety and depression. For both sub-scales, the HADS score can take on values between 0 and 21, reflecting increasing anxiety and depression symptom-loads. Values between 8 and 10 are judged to be signs of clinical anxiety/depression levels, and values above 10 as indicators of acute need for professional treatment. A recent literature review demonstrated the HADS instrument’s effective case-finding properties for anxiety and depression, both in primary care patient populations and in hospital settings [34]. Assessment of anxiety and depression via the HADS sub-scales has been shown to be more accurate than the diagnostic skills of general practitioners, and thus a suitable surrogate for diagnosis [35]. An optimal cut-off value of 8 has been determined for dichotomous categorization of the original score values, with the aim of identifying persons suffering from anxiety or depressive disorders: Using the recommended cut-off value of ≥8 for HADS-Anxiety and HADS-Depression, 76% and 87%, respectively, are classified correctly from a clinical perspective [36].

### Analyses

Descriptive analyses of the data on perceived health—the SF-12 physical and mental sub-scales—mental health—HADS scores for depression and anxiety—socio-demographic characteristics, health status, and health behavior were carried out for the total sample (n = 365), reporting the mean with SD for numerical data and percentages for categorical data. The reported percentages refer to the number of cases available per variable. Sample means of SF-12 and HADS values were compared with their respective reference values using t-tests with one-sided hypotheses.

To assess which of the mental health, socio-demographic, health status, and health behavior variables bore significant relationships to perceived health in our sample of long-term unemployed individuals, the relationship between each of these potential explanatory variables and perceived health was first examined in bivariate analyses: SF-12 physical and mental sub-scales were compared across categories of the individual explanatory variables, using Mann–Whitney U tests for comparisons of two groups and Kruskal-Wallis tests for comparison of three or more groups, for significance testing (p < 0.05).

In order to assess the combined effect of the mental health, socio-demographic, health status, and health behavior variables after adjusting for age and sex, multiple linear regression models using perceived physical or mental health as a dependent variable were fitted to the data.

The decision to use the SF-12 physical and mental component scores as dependent variables was based partly on a theoretical presumption, and on previous findings detailing the importance of self-perceived health in unemployment research [19-22]. The independent variables for which numerical data was available—age, HADS-Depression, HADS-Anxiety, BMI, and AUDIT-C—were first assessed graphically in partial regression plots adjusted for age and sex, to judge the adequacy of a linear term. To avoid problems with multicollinearity, scatterplots and Pearson correlation coefficients were used to identify potentially problematic pairings of variables. For the two variables expected to show the greatest correlation, HADS-Depression and HADS-Anxiety, a combined categorical variable, “problematic mental health status” (HADS-Anxiety > =8 and/or HADS-Depression > = 8) used in previous publications [35], was considered as an alternative. The other categorical variables included as independent variables in the linear regression were gender, educational level, inclusion in a steady relationship, duration of unemployment, personal migration experience, setting, hypertension, smoking habits, and level of physical activity.

The model-building approach used was a forward selection algorithm using SAS PROC GLMSELECT with a significance level of 0.10 as the entry criterion. Age and gender were fitted to the model. The resultant model from the forward selection was then refitted with SAS PROC GLM, and graphical model diagnostics were carried out. These included checks for outliers and influential observations, assessments of normality and the homoscedasticity of errors.

In a final step, the model was refitted in SAS PROC combined with the integration of a random intercept term on the recruitment center level. This was done in order to take into account any correlation that might be present in the data due to the clustered sampling of individuals. A likelihood ratio test approach comparing this model with the fixed effects model—in which the p-values were halved to avoid overly conservative tests due to variance boundary problems, cf. [37]—was used to determine the necessity of integrating the random effect. All analyses were carried out in SAS for Windows 9.2.

## Results

### Sample description

Table 1 shows the characteristics of the respondents completing the baseline questionnaire. The mean age of respondents was 43.7 years; 58% were female; over 33% had a low level of education, defined as fewer than 10 years formal schooling. Thirty-six percent reported on their personal migration experience, and more than 50% of the sample population had been unemployed for five or more years; 20% of respondents had never worked in Germany. More than 60% were not living with a partner in a steady relationship. Hypertension, according to our definition, was present in 34% of the sample. Twenty-nine percent of respondents were classified as obese (BMI ≥30). Fifty-four percent were current smokers, and 23% were determined to be exhibiting high-risk alcohol consumption patterns. A low- or medium-level of physical activity—exercising less than three times per week—was reported by 75% of respondents.

**Table 1:** Sample description

**Characteristic**		**Total sample (n=365)**
**Demographic variables**		
Age (years)	mean (SD)	43.7 (11.1)
Women	n (%)	211 (57.8)
School years	mean (SD)	10.6 (1.9) [5]
School years, categorized		[5]
< 10 years	n (%)	120 (33.3)
10-11 years	n (%)	116 (32.2)
>= 12 years	n (%)	124 (34.4)
**Social variables**		
Living in steady relationship	n (%)	134 (37.2) [5]
Duration of unemployment		[12]
< 5 years	n (%)	106 (30.0)
>= 5 years	n (%)	178 (50.4)
never worked in Germany	n (%)	69 (19.6)
Migration experience	n (%)	132 (36.2)
Setting job centre (vs. secondary labour market)	n (%)	76 (20.8%)
**Perceived health variables**		
PCS (SF-12)	mean (SD)	44.6 (10.0) [1]
MCS (SF-12)	mean (SD)	44.0 (11.3) [1]
Depression (HADS summary score)	mean (SD)	6.4 (4.2)[2]
HADS >= 11	n (%)	67 (18.5)
8<=HADS<11	n (%)	66 (18.2)
HADS < 8	n (%)	230 (63.4)
Anxiety (HADS summary score)	mean (SD)	7.3 (4.2) [3]
HADS >= 11	n (%)	89 (24.6)
8<=HADS<11	n (%)	82 (22.7)
HADS < 8	n (%)	191 (52.8)
Problematic mental health status (HADS Depression >= 8 and/or HADS Anxiety >=8)	n (%)	198 (54.6) [2]
**Health status variables (risk factors)**		
Blood pressure (systol.)	mean (SD)	126.0 (19.8) [6]
Blood pressure (diast.)	mean (SD)	85.5 (12.7) [6]
Hypertonus (>140/90)	n (%)	121 (33.7) [6]
Body Mass Index (BMI)	mean (SD)	27.8 (6.5) [6]
BMI >= 30	n (%)	104 (29.0)
25 <= BMI < 30	n (%)	116 (32.3)
18.5 <= BMI < 25	n (%)	132 (36.8)
BMI < 18.5	n (%)	7 (2.0)
**Health behaviour variables (risk factors)**		
Physical activity		[9]
low/medium	n (%)	267 (75.0)
high (at least three times/week)	n (%)	89 (25.0)
Smoking behaviour		[6]
Smokers	n (%)	193 (53.8)
Never smoked	n (%)	119 (33.2)
Stopped smoking	n (%)	47 (13.1)
Alcohol consumption (AUDIT-C screening test)		[7]
no consumption (♂: 0 ♀: 0)	n (%)	116 (32.4)
moderate consumption (♂: 1–4; ♀: 1–3)	n (%)	160 (44.7)
risk consumption (♂: ≥ 5; ♀: ≥ 4)	n (%)	82 (22.9)

* Numbers in square brackets denote numbers of missing values.

Anxiety disorders, defined by HADS-Anxiety values ≥8, were observed in 47% of participants, and 37% showed evidence of depressive disorders, defined by HADS-Depression values ≥8. Both men and women showed significantly increased mean levels on the HADS sub-scales—HADS-Anxiety: 6.6 and 7.9 respectively; HADS-Depression: 6.5 and 6.3, respectively—as compared to reference values (p < 0.0001) [38]. As Table 1 shows, the mean values of the PCS and MCS sub-scales were 44.6 and 44.0 in the sample, overall. As with the HADS sub-scales, both men and women showed significantly reduced PCS and MCS values—men: 44.7 and 45.7 respectively; women: 44.5 and 42.7 respectively—compared to reference values for the general population (p < .0001) [30]. The correlation between PCS and MCS was r = 0.107.

### Factors of perceived health in long-term unemployed

**Figure 1:**
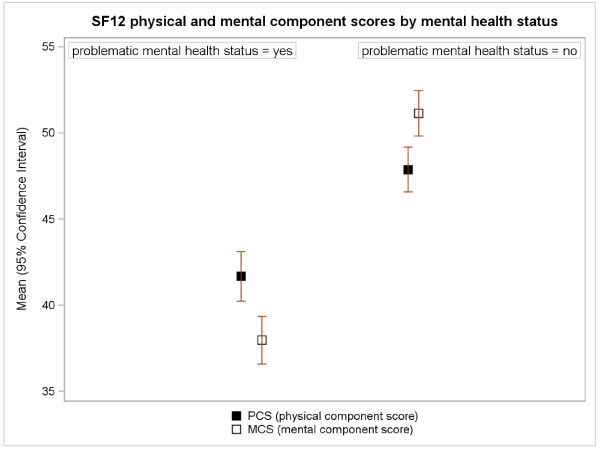
Mean (95% CI) SF-12 scores by problematic health status.

As is illustrated in Figure 1, the mean physical and mental SF-12 scores were significantly lower in the group of participants with problematic mental health statuses (participants with a HADS-Anxiety ≥8 and/or HADS-Depression ≥8). These results were confirmed in the multiple linear regression models presented in Table 2. According to HADS, problematic mental health status was the factor most strongly correlated with both dimensions of perceived health. After adjusting for all other variables in the model, problematic mental health status led to an estimated reduction in the physical and mental SF-12 scores of 4.9 and 12.7 points, respectively. The other independent variables included in the age-sex adjusted final regression model for perceived physical health were BMI, which was negatively associated with perceived physical health; and, setting. After adjusting for all other variables in the model, there was an estimated reduction of 3.2 points in the SF-12 physical component score for unemployed persons recruited from the job centers as opposed to the secondary labor market.

**Table 2:** Predictors of physical and mental SF-12 scores. Results of the multiple linear regression models

**PCS (SF-12)/final model R**^**2**^**=0.20**				**MCS (SF-12)/final model R**^**2**^**=0.36**			
	**Parameter**	**SE**	**p-value**		**Parameter**	**SE**	**p-value**
intercept	46.33	0.978	<0.0001	intercept	50.18	0.943	<0.0001
sex: male	0.44	0.982	0.6543	sex: male	1.48	1.002	0.1411
AGE	−0.11	0.056	0.0519	AGE	0.14	0.051	0.0069
AGE^2^	0.01	0.004	0.0104	setting: job centre	−3.61	1.413	0.0111
setting: job centre	−3.19	1.378	0.0210	problematic mental health status: yes	−12.71	1.010	<0.0001
problematic mental health status: yes	−4.93	0.984	<0.0001	physical activity: high	3.41	1.163	0.0036
BMI	−0.24	0.074	0.0016				

AGE: linear age term, AGE^2^: quadratic age term.

Variables AGE and BMI centered around grand mean (AGE: 44, BMI: 28).

In the age-sex adjusted final regression model for perceived mental health, the independent variables included alongside a problematic mental health status as defined by HADS were setting and level of physical activity. After adjusting for all other variables in the model, setting was included with an estimated reduction of 3.6 points in the SF-12 mental component score for unemployed persons recruited from job centers as opposed to the secondary labor market. After adjusting for all other variables in the model, physical activity accounted for an estimated increase of 3.4 points in the SF-12 mental component score for unemployed persons reporting high levels of physical activity as opposed to those reporting only low- to medium-levels of physical activity. As is explained in Table 2, the factors included in the respective linear regression models together explained 20% of variation in perceived physical health, and 36% of variation in perceived mental health.

In the bivariate analyses only, duration of unemployment was also strongly associated with perceived physical health: Participants with a history of five or more years of unemployment reported lower levels of physical health (PCS = 42.9; SD = 10.3). Alcohol consumption patterns were also significantly associated with perceived physical health in the bivariate analyses, with better perceived health among persons reporting moderate alcohol consumption as opposed to both persons assessed as high-risk consumers and those reporting no alcohol consumption at all.

## Discussion

### Main results

Differences in perceived mental and physical health within our sample of long-term unemployed individuals were significantly associated with problematic mental health statuses, setting-specific effects, and certain health status and behavior variables, such as BMI and participants’ typical level of physical activity. These variables together explained 20% of the variance in perceived physical health and 36% of the variance in perceived mental health in an age-sex adjusted model. Although these findings are preliminary and methodologically limited, our results argue in favor of increased efforts to develop specific interventions for unemployed individuals at high risk of low perceived health. As we have shown, a relevant subgroup of long-term unemployed individuals are affected by reduced mental health, elevated BMI and/or low levels of physical activity, and are exposed to settings of the social welfare system offering less social support.

### Limitations and strengths

Prior to proceeding with the discussion of the results of this study, certain important methodological issues must be discussed.

A major limitation of this study is that it is a cross-sectional survey, making causal interpretations hazardous. We thus refer to associations between variables, instead of emphasizing the concept of determinants. Our study does not allow for conclusions on the effects of unemployment on health and is only a descriptive analysis of variable patterns related to perceived health in a specific group of long-term unemployed persons in Germany. Second, a valid estimate of the true response was hindered by several selection biases which restrict the applicability of the findings: besides a health-related selection into unemployment, a selection bias due to voluntary participation and the non-randomized selection of two settings of welfare-to-work organizations is likely. Study participants may constitute a subgroup of more motivated long-term unemployed persons, and so a generalization of the findings is consequently not valid. Third, as self-reported health data may lead to under- or over-estimation of the real health status of a given individual, we cannot objectively describe the health status of our sample. Moreover, the list of symptoms and conditions is not complete. The use of perceived health as a dependent variable in our model is methodologically grounded in recent studies [20-22], but remains to be validated in longitudinal approaches. Finally, the number of participants in the two settings was unequal (setting Munich, n = 289; setting Hanover, n = 76) and the respective settings appear to be geared toward the needs of distinct sub-groups of the long-term unemployed.

The primary advantage of our study stems from the fact that it is setting-based, and that we analyzed a target group rarely addressed by health research, mainly due to the expected non-compliance of individuals to study conditions. Apart from a reasonable number of participants, it was also possible to include and better characterize important subgroups, such as participants with personal migration experiences. The use of validated, standardised questionnaires to assess perceived health, anxiety, and depression is a further strength of this study. The SF-12 questionnaire was developed for use in general populations (i.e. not for express use in clinical settings), and is traditionally used to measure functional health status. It has also been used in unemployment research previously [cf., 20, 21, 22], where patients’ perception of health was discussed as an important obstacle to a successful return to work. The HADS questionnaire is a clinically validated instrument which has also been used in unemployment research [21,36]. Furthermore, health behavior and socio-economic variables relevant for both health and the ability to work were documented.

### Interpretation

Compared to general population data and to other reports on the health status of unemployed persons in epidemiological studies [21] or intervention trials [22], participants in this cross-sectional study showed signs of reduced health with respect to the following parameters:

1) Perceived health in our sample differed significantly not only from the norm values of the general population, but also from findings in other unemployment studies. The mean value of perceived physical health in our group of long-term unemployed persons was 44.6 (SD = 10.0). As an illustrative example, a recently published Norwegian sample of unemployed persons [21] yielded a value of 49.62. The difference in perceived mental health was even more acute, with a mean value in our group of 44.0 (SD = 11.3), as opposed to 51.23 in the Norwegian sample. A five point difference in the SF-12 scores is considered indicative of a clinically relevant change or difference [30]. The description of the Norwegian sample did not provide additional information on the length of unemployment of their participants, although the authors did note that long-term sickness absentees were analyzed separately. The health differences between the two samples illustrate the need for more differentiation and subgroup analyses in unemployment research.

2) The low levels of perceived health observed in this study, with particularly pronounced deviations from the reference value for the mental SF-12 sub-score, are mirrored by HADS-Anxiety and HADS-Depression scores which are also significantly lower than the corresponding reference values. Compared to other samples of unemployed persons, the HADS results differ according to the HADS dimension: The mean value of the HADS-Depression score in our sample was 6.4 (SD = 4.2), as compared to 4.9 (SD = 3.0) in a sample of younger unemployed persons (mean age 29 years) in Germany who had experienced several instances of unemployment [36]. The mean value of the HADS-Anxiety sub-scale in our sample was 7.3 (SD = 4.2), similar to the 7.44 (SD = 3.32) reported in the younger cohort of unemployed persons [36]. In a meta-analysis by Paul and Moser [14], the average number of persons with psychological problems among the unemployed was 34%, whereas in our sample of very-long-term unemployed persons 55% showed evidence of depression and/or anxiety disorders.

3) The prevalence of obesity in our study was 29%, as compared to 11.7% in women and 12.9% in men in the general German population [39]. In a Dutch multidisciplinary health program for unemployed persons with health complaints, 29.4% of individuals were classed as obese [22]. In our sample, 34% of participants were shown to be suffering from hypertension, whereas in the general population the one-year-prevalence of hypertension was only 7.3% among women and 9.8% among men [39]. Health-risk behavior was also widespread in our sample: 54% of participants were smokers, whereas the prevalence rates for smoking in the general German population stand at 33.4% among women and 42.2% among men [39].

The results of the linear regression models indicate that mental and physical perceived health in long-term unemployed persons are both significantly associated with mental health status. Not surprisingly, mental health status as measured by HADS was the main predictor of perceived mental health as measured by the SF-12 mental component score (p < 0.0001). The fact that mental health status as measured by HADS was also the main predictor of perceived physical health is consistent with the findings of the Whitehall II study, which demonstrated a moderate correlation between mental and physical health among people with a low socioeconomic status [40]. Schutgens et al. [22] conclude that “perceived health may be influenced by cognitions, for example the way people cope with their health problems”, which might explain the impact of mental health status on reduced PCS and MCS scores in our sample.

Perceived mental health was also significantly associated with individuals’ level of physical activity, providing some evidence of the importance of physical activity to this particular group. Previous studies have reported contradictory evidence concerning the impact of physical activity interventions on the health status of the unemployed: A Dutch health program, focused on changing health behaviour—e.g. by offering sessions of physical activity—and teaching coping strategies for health complaints, failed to show positive effects on perceived health [41], whereas Watson and colleagues [42] reported some positive effects stemming from physical activity interventions in combination with cognitive behavioral interventions among unemployed persons.

The fact that women reported significantly lower levels of perceived mental health in our sample of long-term unemployed persons supports calls for more gender-focused research into the long-term health effects of unemployment. In the meta-analysis of McKee-Ryan et al. [4], the changing role of women in the workplace is discussed as a potentially plausible explanation for the differences in association between health and unemployment among men and women.

Interestingly, the setting of the welfare to work organization (the Hanover job centers as opposed to the non-profit organizations of the secondary labor market in Munich) appeared to play a more important role in differentiating between unemployed persons with different levels of perceived health than did the personal socio-demographic characteristics of participants. The exact reasons for the differences between the two settings can only be hypothesized: In Hanover, the average age of participants was higher than in Munich; the setting variable remained significant, however, even after adjusting for age and gender. From a salutogenic perspective the setting in Munich offers more structure, increased levels of social support, and a better perspective for their clients than does the Hanover job center. These setting specific differences may contribute to improvements in perceived health, especially in a subgroup possessed of severe mental health problems. An additional difference between settings is the fact that the labor market in Hanover is less dynamic than that in Munich. The results concerning differences in setting should be interpreted with caution, however, as the two settings may address different sub-groups of unemployed persons, and both the recruiting process and requirement of voluntary participation may have biased the results.

Surprisingly, we could not validate the influence of some factors mentioned in the literature which might have influenced the perceived health of the individuals comprising our sample. According to the meta-analysis of McKee-Ryan and colleagues, sample type—in particular, recent graduates as compared to mature unemployed persons—is an important predictor of mental health among the unemployed [4]. In contrast, educational level and personal migration experience were not found to contribute to the final regression models used in this study, and did not show significant associations with perceived health in the bivariate analyses of the cross-sectional analyses presented here.

In Paul and Moser’s meta-analyses [14], a curvilinear relationship between unemployment duration and mental health was identified, with a stabilization of mental health during the second year of unemployment and increasing psychological problems among very-long-term unemployed persons. In our selected sample, more than 50% of participants had been unemployed for five or more years. The bivariate analyses of the SF-12 mental sub-score did not show significant differences between the different categories of unemployment duration. The equivalent analysis of differences in the physical health score, however, showed that participants with a history of five or more years of unemployment reported significantly reduced physical health scores.

Although the regression analyses identified important factors associated with self-perceived health in our sample, the findings also suggest that additional factors not measured in our study may explain key differences in perceived health among the long-term unemployed. The fact that only 20% of the variation in the SF-12 physical component scores was explained by the regression model—which included age, gender, setting, and BMI, along with mental health status according to HADS—illustrates the need to gain a better understanding of those factors other than mental health status or setting effects influencing the perceived health of long-term unemployed persons. Due to the fact that only a fairly select few health variables were assessed in our analyses, our findings here are preliminary and limited. Additional methodologically elaborated research, particularly longitudinal designs, will help elucidate the relationship between perceived health, long-term unemployment, and both identified and as-yet-unidentified explanatory factors.

## Conclusions

This study has shown that research on the impact of long-term unemployment must have a stronger focus on identifying factors associated with differences in self-perceived health, which has been identified as a key factor for a successful return to work in previous studies. Evidence-based indicators for determinants of perceived health would allow for the design of targeted intervention programs aimed at improving perceived health. These efforts would be a prerequisite to increasing the re-employment chances of the long-term unemployed.

## Competing interests

The authors have no competing interests and declare no financial interests.

## Authors’ contributions

HL, MH, KH, KS, HG, and PA made substantial contributions to conception and design, acquisition, analysis, and interpretation of data. HL, MH, and KS were involved in drafting the manuscript. All authors read and approved the final manuscript.

## Pre-publication history

The pre-publication history for this paper can be accessed here:

http://www.biomedcentral.com/1471-2458/12/485/prepub
